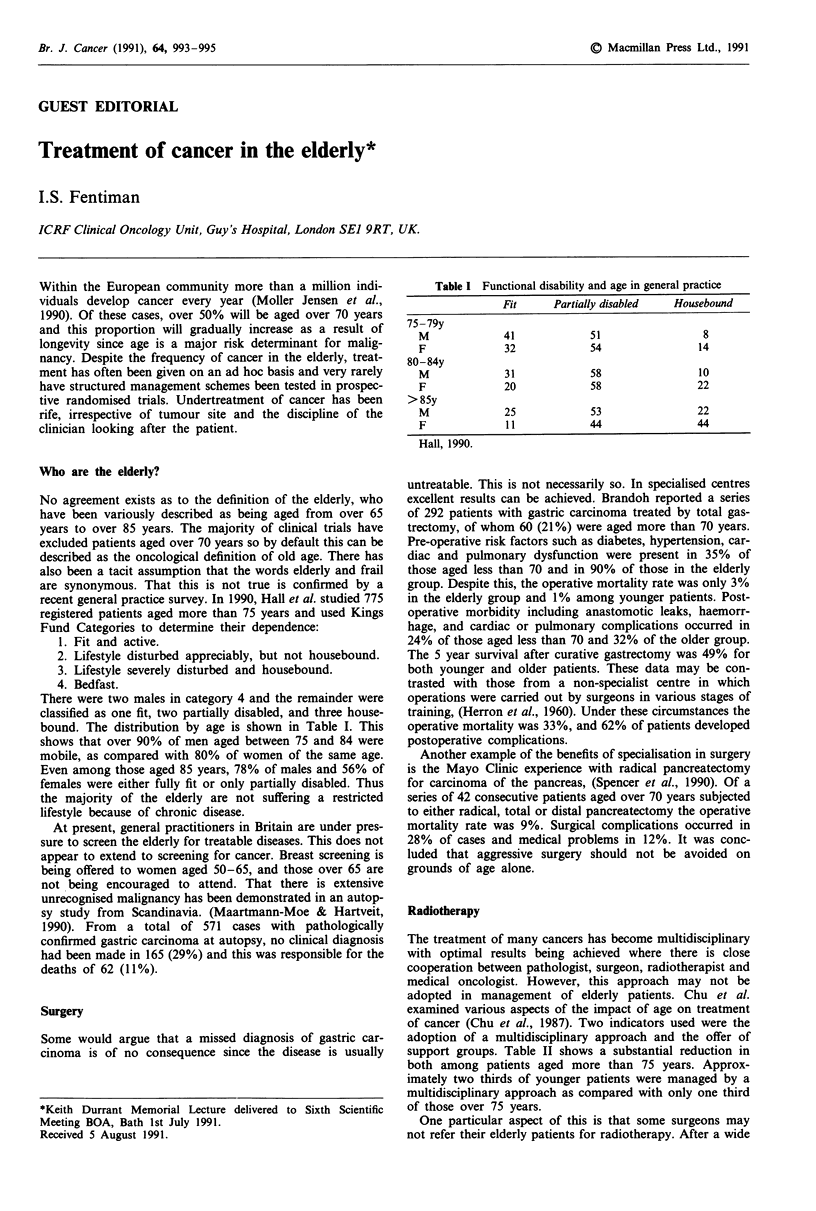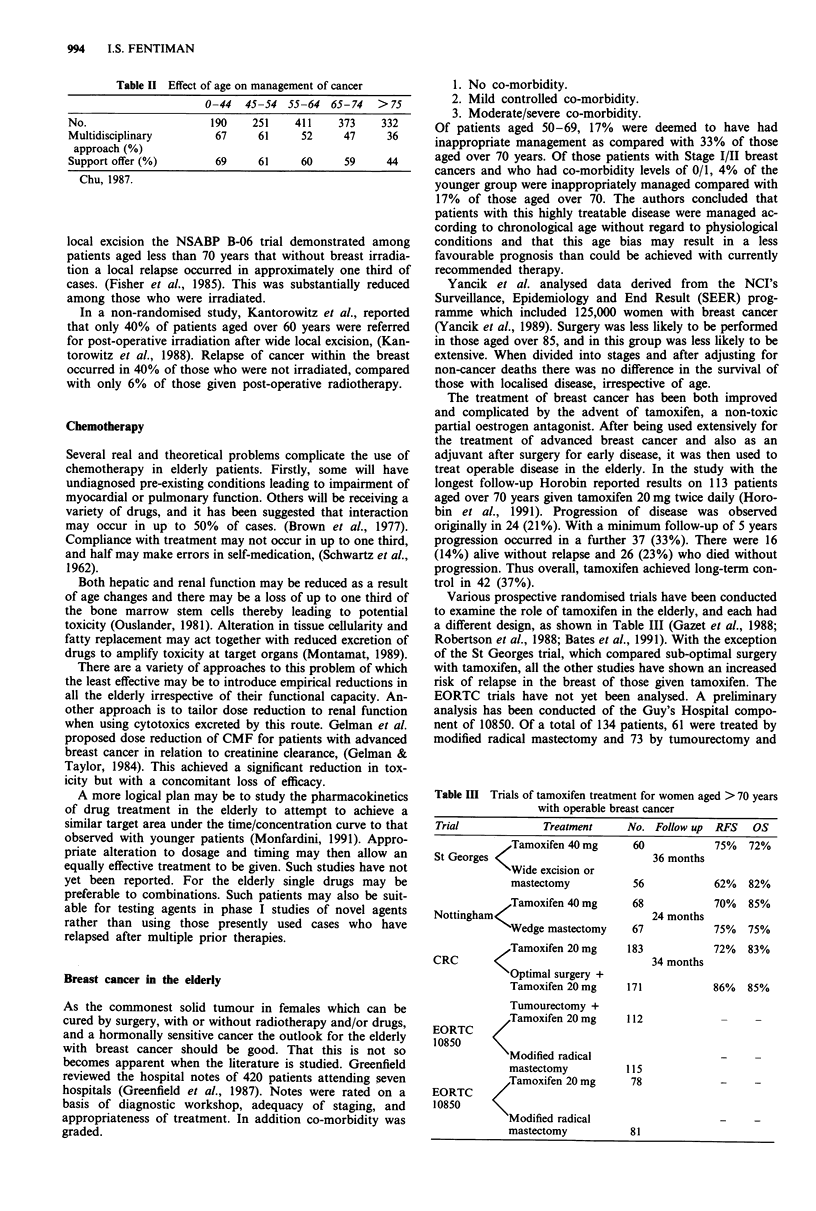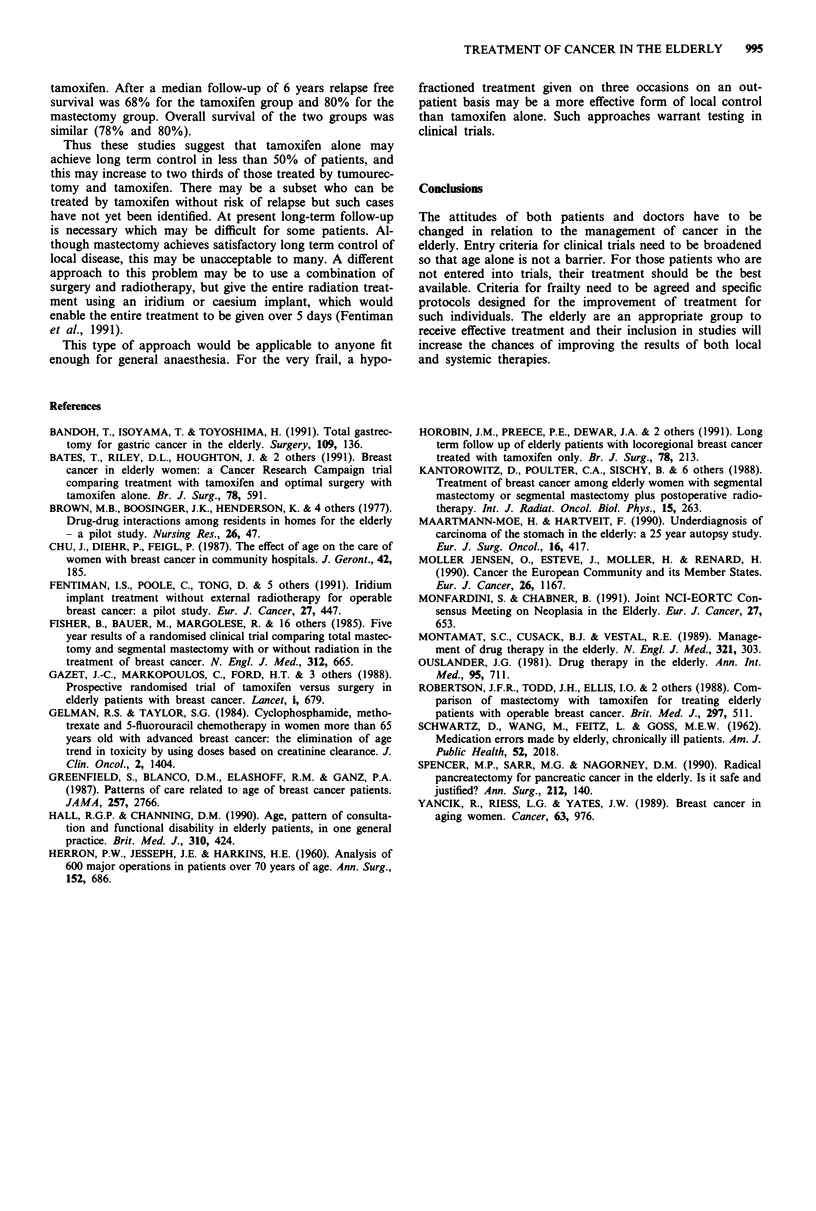# Treatment of cancer in the elderly.

**DOI:** 10.1038/bjc.1991.453

**Published:** 1991-12

**Authors:** I. S. Fentiman


 © Macmillan Press Ltd., 1991

GUEST EDITORIAL

### Treatment of cancer in the elderly*

I.S. Fentiman

*ICRF Clinical Oncology Unit, Guy's Hospital, London SE1 9RT, UK.*

Within the European community more than a million individuals develop cancer every year (Moller Jensen *et al.*, 1990). Of these cases, over 50% will be aged over 70 years and this proportion will gradually increase as a result of longevity since age is a major risk determinant for malignancy. Despite the frequency of cancer in the elderly, treatment has often been given on an ad hoc basis and very rarely have structured management schemes been tested in prospective randomised trials. Undertreatment of cancer has been rife, irrespective of tumour site and the discipline of the clinician looking after the patient.

### Who are the elderly?

No agreement exists as to the definition of the elderly, who have been variously described as being aged from over 65 years to over 85 years. The majority of clinical trials have excluded patients aged over 70 years so by default this can be described as the oncological definition of old age. There has also been a tacit assumption that the words elderly and frail are synonymous. That this is not true is confirmed by a recent general practice survey. In 1990, Hall *et al.* studied 775 registered patients aged more than 75 years and used Kings Fund Categories to determine their dependence:

1. Fit and active.
2. Lifestyle disturbed appreciably, but not housebound.
3. Lifestyle severely disturbed and housebound.
4. Bedfast.

There were two males in category 4 and the remainder were classified as one fit, two partially disabled, and three housebound. The distribution by age is shown in Table I. This shows that over 90% of men aged between 75 and 84 were mobile, as compared with 80% of women of the same age. Even among those aged 85 years, 78% of males and 56% of females were either fully fit or only partially disabled. Thus the majority of the elderly are not suffering a restricted lifestyle because of chronic disease.

At present, general practitioners in Britain are under pressure to screen the elderly for treatable diseases. This does not appear to extend to screening for cancer. Breast screening is being offered to women aged 50-65, and those over 65 are not being encouraged to attend. That there is extensive unrecognised malignancy has been demonstrated in an autopsy study from Scandinavia. (Maartmann-Moe & Hartveit, 1990). From a total of 571 cases with pathologically confirmed gastric carcinoma at autopsy, no clinical diagnosis had been made in 165 (29%) and this was responsible for the deaths of 62 (11%).

### Surgery

Some would argue that a missed diagnosis of gastric carcinoma is of no consequence since the disease is usually untreatable. This is not necessarily so. In specialised centres excellent results can be achieved. Brandoh reported a series of 292 patients with gastric carcinoma treated by total gastrectomy, of whom 60 (21%) were aged more than 70 years. Pre-operative risk factors such as diabetes, hypertension, cardiac and pulmonary dysfunction were present in 35% of those aged less than 70 and in 90% of those in the elderly group. Despite this, the operative mortality rate was only 3% in the elderly group and 1% among younger patients. Postoperative morbidity including anastomotic leaks, haemorrhage, and cardiac or pulmonary complications occurred in 24% of those aged less than 70 and 32% of the older group. The 5 year survival after curative gastrectomy was 49% for both younger and older patients. These data may be contrasted with those from a non-specialist centre in which operations were carried out by surgeons in various stages of training, (Herron *et al.*, 1960). Under these circumstances the operative mortality was 33%, and 62% of patients developed postoperative complications.

**Table I** Functional disability and age in general practice

| | *Fit* | *Partially disabled* | *Housebound* |
|---|---|---|---|
| 75-79y | | | |
| M | 41 | 51 | 8 |
| F | 32 | 54 | 14 |
| 80-84y | | | |
| M | 31 | 58 | 10 |
| F | 20 | 58 | 22 |
| >85y | | | |
| M | 25 | 53 | 22 |
| F | 11 | 44 | 44 |

Hall, 1990.

Another example of the benefits of specialisation in surgery is the Mayo Clinic experience with radical pancreatectomy for carcinoma of the pancreas, (Spencer *et al.*, 1990). Of a series of 42 consecutive patients aged over 70 years subjected to either radical, total or distal pancreatectomy the operative mortality rate was 9%. Surgical complications occurred in 28% of cases and medical problems in 12%. It was concluded that aggressive surgery should not be avoided on grounds of age alone.

### Radiotherapy

The treatment of many cancers has become multidisciplinary with optimal results being achieved where there is close cooperation between pathologist, surgeon, radiotherapist and medical oncologist. However, this approach may not be adopted in management of elderly patients. Chu *et al.* examined various aspects of the impact of age on treatment of cancer (Chu *et al.*, 1987). Two indicators used were the adoption of a multidisciplinary approach and the offer of support groups. Table II shows a substantial reduction in both among patients aged more than 75 years. Approximately two thirds of younger patients were managed by a multidisciplinary approach as compared with only one third of those over 75 years.

One particular aspect of this is that some surgeons may not refer their elderly patients for radiotherapy. After a wide

*Keith Durrant Memorial Lecture delivered to Sixth Scientific Meeting BOA, Bath 1st July 1991.

Received 5 August 1991.

Table II Effect of age on management of cancer

| | *0–44* | *45–54* | *55–64* | *65–74* | *>75* |
|---|---|---|---|---|---|
| No. | 190 | 251 | 411 | 373 | 332 |
| Multidisciplinary approach (%) | 67 | 61 | 52 | 47 | 36 |
| Support offer (%) | 69 | 61 | 60 | 59 | 44 |

Chu, 1987.

local excision the NSABP B-06 trial demonstrated among patients aged less than 70 years that without breast irradiation a local relapse occurred in approximately one third of cases. (Fisher *et al.*, 1985). This was substantially reduced among those who were irradiated.

In a non-randomised study, Kantorowitz *et al.*, reported that only 40% of patients aged over 60 years were referred for post-operative irradiation after wide local excision, (Kantorowitz *et al.*, 1988). Relapse of cancer within the breast occurred in 40% of those who were not irradiated, compared with only 6% of those given post-operative radiotherapy.

### Chemotherapy

Several real and theoretical problems complicate the use of chemotherapy in elderly patients. Firstly, some will have undiagnosed pre-existing conditions leading to impairment of myocardial or pulmonary function. Others will be receiving a variety of drugs, and it has been suggested that interaction may occur in up to 50% of cases. (Brown *et al.*, 1977). Compliance with treatment may not occur in up to one third, and half may make errors in self-medication, (Schwartz *et al.*, 1962).

Both hepatic and renal function may be reduced as a result of age changes and there may be a loss of up to one third of the bone marrow stem cells thereby leading to potential toxicity (Ouslander, 1981). Alteration in tissue cellularity and fatty replacement may act together with reduced excretion of drugs to amplify toxicity at target organs (Montamat, 1989).

There are a variety of approaches to this problem of which the least effective may be to introduce empirical reductions in all the elderly irrespective of their functional capacity. Another approach is to tailor dose reduction to renal function when using cytotoxics excreted by this route. Gelman *et al.* proposed dose reduction of CMF for patients with advanced breast cancer in relation to creatinine clearance, (Gelman & Taylor, 1984). This achieved a significant reduction in toxicity but with a concomitant loss of efficacy.

A more logical plan may be to study the pharmacokinetics of drug treatment in the elderly to attempt to achieve a similar target area under the time/concentration curve to that observed with younger patients (Monfardini, 1991). Appropriate alteration to dosage and timing may then allow an equally effective treatment to be given. Such studies have not yet been reported. For the elderly single drugs may be preferable to combinations. Such patients may also be suitable for testing agents in phase I studies of novel agents rather than using those presently used cases who have relapsed after multiple prior therapies.

### Breast cancer in the elderly

As the commonest solid tumour in females which can be cured by surgery, with or without radiotherapy and/or drugs, and a hormonally sensitive cancer the outlook for the elderly with breast cancer should be good. That this is not so becomes apparent when the literature is studied. Greenfield reviewed the hospital notes of 420 patients attending seven hospitals (Greenfield *et al.*, 1987). Notes were rated on a basis of diagnostic workshop, adequacy of staging, and appropriateness of treatment. In addition co-morbidity was graded.

1. No co-morbidity.
2. Mild controlled co-morbidity.
3. Moderate/severe co-morbidity.

Of patients aged 50–69, 17% were deemed to have had inappropriate management as compared with 33% of those aged over 70 years. Of those patients with Stage I/II breast cancers and who had co-morbidity levels of 0/1, 4% of the younger group were inappropriately managed compared with 17% of those aged over 70. The authors concluded that patients with this highly treatable disease were managed according to chronological age without regard to physiological conditions and that this age bias may result in a less favourable prognosis than could be achieved with currently recommended therapy.

Yancik *et al.* analysed data derived from the NCI's Surveillance, Epidemiology and End Result (SEER) programme which included 125,000 women with breast cancer (Yancik *et al.*, 1989). Surgery was less likely to be performed in those aged over 85, and in this group was less likely to be extensive. When divided into stages and after adjusting for non-cancer deaths there was no difference in the survival of those with localised disease, irrespective of age.

The treatment of breast cancer has been both improved and complicated by the advent of tamoxifen, a non-toxic partial oestrogen antagonist. After being used extensively for the treatment of advanced breast cancer and also as an adjuvant after surgery for early disease, it was then used to treat operable disease in the elderly. In the study with the longest follow-up Horobin reported results on 113 patients aged over 70 years given tamoxifen 20 mg twice daily (Horobin *et al.*, 1991). Progression of disease was observed originally in 24 (21%). With a minimum follow-up of 5 years progression occurred in a further 37 (33%). There were 16 (14%) alive without relapse and 26 (23%) who died without progression. Thus overall, tamoxifen achieved long-term control in 42 (37%).

Various prospective randomised trials have been conducted to examine the role of tamoxifen in the elderly, and each had a different design, as shown in Table III (Gazet *et al.*, 1988; Robertson *et al.*, 1988; Bates *et al.*, 1991). With the exception of the St Georges trial, which compared sub-optimal surgery with tamoxifen, all the other studies have shown an increased risk of relapse in the breast of those given tamoxifen. The EORTC trials have not yet been analysed. A preliminary analysis has been conducted of the Guy's Hospital component of 10850. Of a total of 134 patients, 61 were treated by modified radical mastectomy and 73 by tumourectomy and

Table III Trials of tamoxifen treatment for women aged >70 years with operable breast cancer

| *Trial* | *Treatment* | *No.* | *Follow up* | *RFS* | *OS* |
|---|---|---|---|---|---|
| St Georges | Tamoxifen 40 mg | 60 | 36 months | 75% | 72% |
| | Wide excision or mastectomy | 56 | | 62% | 82% |
| Nottingham | Tamoxifen 40 mg | 68 | 24 months | 70% | 85% |
| | Wedge mastectomy | 67 | | 75% | 75% |
| CRC | Tamoxifen 20 mg | 183 | 34 months | 72% | 83% |
| | Optimal surgery + Tamoxifen 20 mg | 171 | | 86% | 85% |
| EORTC 10850 | Tumourectomy + Tamoxifen 20 mg | 112 | | – | – |
| | Modified radical mastectomy | 115 | | – | – |
| EORTC 10850 | Tamoxifen 20 mg | 78 | | – | – |
| | Modified radical mastectomy | 81 | | – | – |

tamoxifen. After a median follow-up of 6 years relapse free survival was 68% for the tamoxifen group and 80% for the mastectomy group. Overall survival of the two groups was similar (78% and 80%).

Thus these studies suggest that tamoxifen alone may achieve long term control in less than 50% of patients, and this may increase to two thirds of those treated by tumourectomy and tamoxifen. There may be a subset who can be treated by tamoxifen without risk of relapse but such cases have not yet been identified. At present long-term follow-up is necessary which may be difficult for some patients. Although mastectomy achieves satisfactory long term control of local disease, this may be unacceptable to many. A different approach to this problem may be to use a combination of surgery and radiotherapy, but give the entire radiation treatment using an iridium or caesium implant, which would enable the entire treatment to be given over 5 days (Fentiman *et al.*, 1991).

This type of approach would be applicable to anyone fit enough for general anaesthesia. For the very frail, a hypofractioned treatment given on three occasions on an outpatient basis may be a more effective form of local control than tamoxifen alone. Such approaches warrant testing in clinical trials.

### Conclusions

The attitudes of both patients and doctors have to be changed in relation to the management of cancer in the elderly. Entry criteria for clinical trials need to be broadened so that age alone is not a barrier. For those patients who are not entered into trials, their treatment should be the best available. Criteria for frailty need to be agreed and specific protocols designed for the improvement of treatment for such individuals. The elderly are an appropriate group to receive effective treatment and their inclusion in studies will increase the chances of improving the results of both local and systemic therapies.

### References

BANDOH, T., ISOYAMA, T. & TOYOSHIMA, H. (1991). Total gastrectomy for gastric cancer in the elderly. *Surgery*, **109**, 136.

BATES, T., RILEY, D.L., HOUGHTON, J. & 2 others (1991). Breast cancer in elderly women: a Cancer Research Campaign trial comparing treatment with tamoxifen and optimal surgery with tamoxifen alone. *Br. J. Surg.*, **78**, 591.

BROWN, M.B., BOOSINGER, J.K., HENDERSON, K. & 4 others (1977). Drug-drug interactions among residents in homes for the elderly – a pilot study. *Nursing Res.*, **26**, 47.

CHU, J., DIEHR, P., FEIGL, P. (1987). The effect of age on the care of women with breast cancer in community hospitals. *J. Geront.*, **42**, 185.

FENTIMAN, I.S., POOLE, C., TONG, D. & 5 others (1991). Iridium implant treatment without external radiotherapy for operable breast cancer: a pilot study. *Eur. J. Cancer*, **27**, 447.

FISHER, B., BAUER, M., MARGOLESE, R. & 16 others (1985). Five year results of a randomised clinical trial comparing total mastectomy and segmental mastectomy with or without radiation in the treatment of breast cancer. *N. Engl. J. Med.*, **312**, 665.

GAZET, J.-C., MARKOPOULOS, C., FORD, H.T. & 3 others (1988). Prospective randomised trial of tamoxifen versus surgery in elderly patients with breast cancer. *Lancet*, **i**, 679.

GELMAN, R.S. & TAYLOR, S.G. (1984). Cyclophosphamide, methotrexate and 5-fluorouracil chemotherapy in women more than 65 years old with advanced breast cancer: the elimination of age trend in toxicity by using doses based on creatinine clearance. *J. Clin. Oncol.*, **2**, 1404.

GREENFIELD, S., BLANCO, D.M., ELASHOFF, R.M. & GANZ, P.A. (1987). Patterns of care related to age of breast cancer patients. *JAMA*, **257**, 2766.

HALL, R.G.P. & CHANNING, D.M. (1990). Age, pattern of consultation and functional disability in elderly patients, in one general practice. *Brit. Med. J.*, **310**, 424.

HERRON, P.W., JESSEPH, J.E. & HARKINS, H.E. (1960). Analysis of 600 major operations in patients over 70 years of age. *Ann. Surg.*, **152**, 686.

HOROBIN, J.M., PREECE, P.E., DEWAR, J.A. & 2 others (1991). Long term follow up of elderly patients with locoregional breast cancer treated with tamoxifen only. *Br. J. Surg.*, **78**, 213.

KANTOROWITZ, D., POULTER, C.A., SISCHY, B. & 6 others (1988). Treatment of breast cancer among elderly women with segmental mastectomy or segmental mastectomy plus postoperative radiotherapy. *Int. J. Radiat. Oncol. Biol. Phys.*, **15**, 263.

MAARTMANN-MOE, H. & HARTVEIT, F. (1990). Underdiagnosis of carcinoma of the stomach in the elderly: a 25 year autopsy study. *Eur. J. Surg. Oncol.*, **16**, 417.

MOLLER JENSEN, O., ESTEVE, J., MOLLER, H. & RENARD, H. (1990). Cancer the European Community and its Member States. *Eur. J. Cancer*, **26**, 1167.

MONFARDINI, S. & CHABNER, B. (1991). Joint NCI-EORTC Consensus Meeting on Neoplasia in the Elderly. *Eur. J. Cancer*, **27**, 653.

MONTAMAT, S.C., CUSACK, B.J. & VESTAL, R.E. (1989). Management of drug therapy in the elderly. *N. Engl. J. Med.*, **321**, 303.

OUSLANDER, J.G. (1981). Drug therapy in the elderly. *Ann. Int. Med.*, **95**, 711.

ROBERTSON, J.F.R., TODD, J.H., ELLIS, I.O. & 2 others (1988). Comparison of mastectomy with tamoxifen for treating elderly patients with operable breast cancer. *Brit. Med. J.*, **297**, 511.

SCHWARTZ, D., WANG, M., FEITZ, L. & GOSS, M.E.W. (1962). Medication errors made by elderly, chronically ill patients. *Am. J. Public Health*, **52**, 2018.

SPENCER, M.P., SARR, M.G. & NAGORNEY, D.M. (1990). Radical pancreatectomy for pancreatic cancer in the elderly. Is it safe and justified? *Ann. Surg.*, **212**, 140.

YANCIK, R., RIESS, L.G. & YATES, J.W. (1989). Breast cancer in aging women. *Cancer*, **63**, 976.